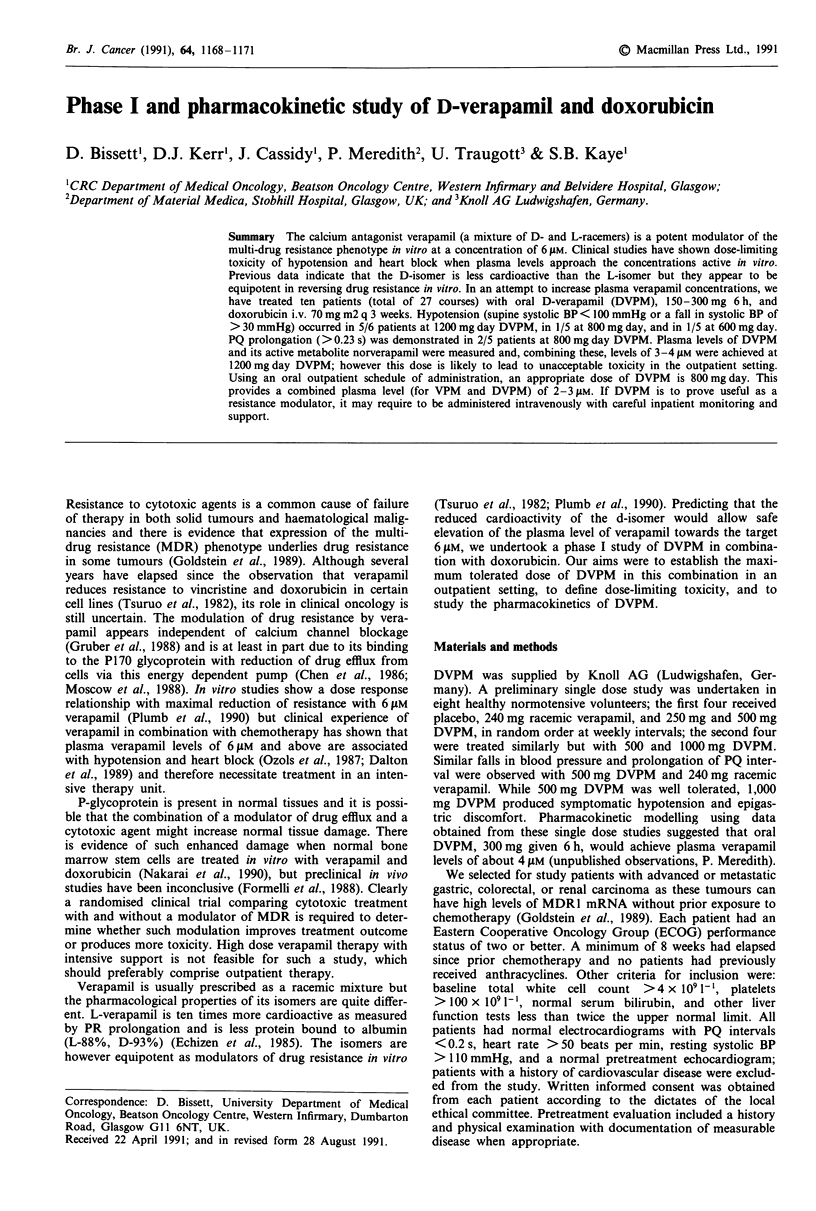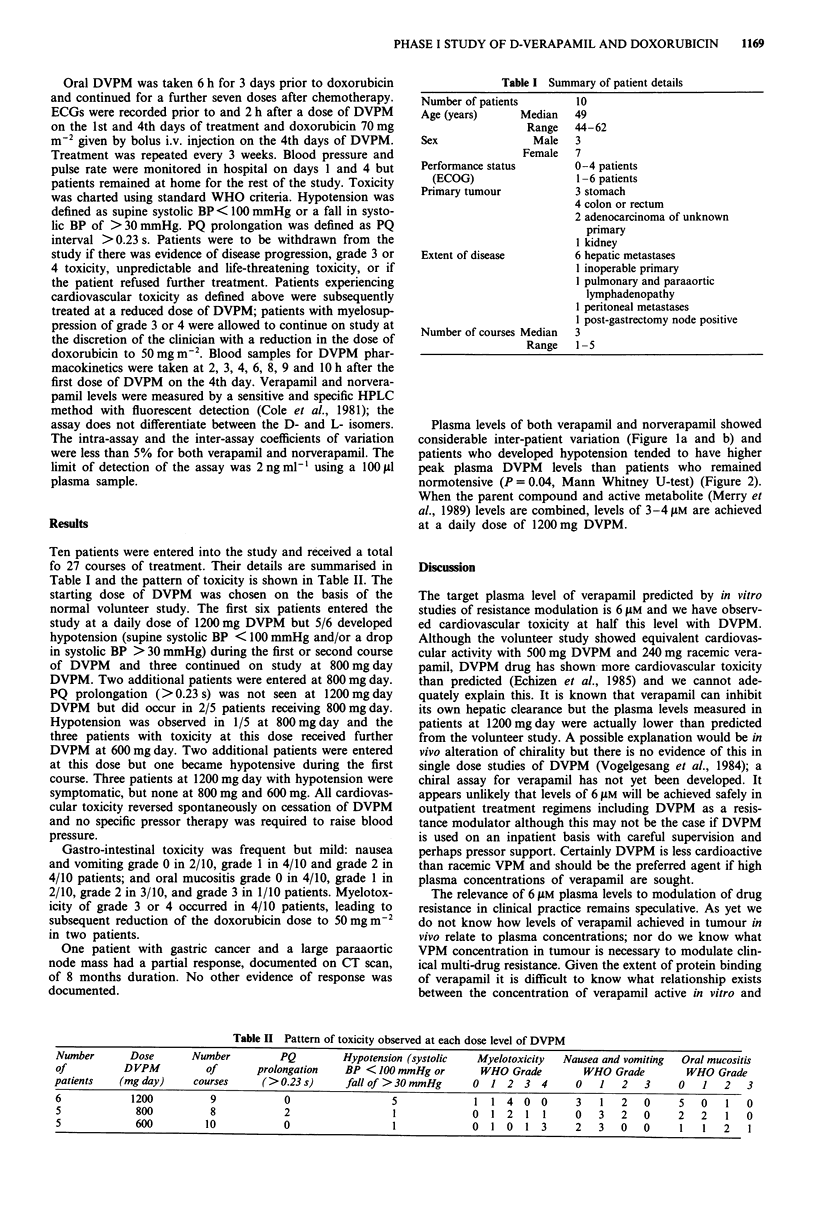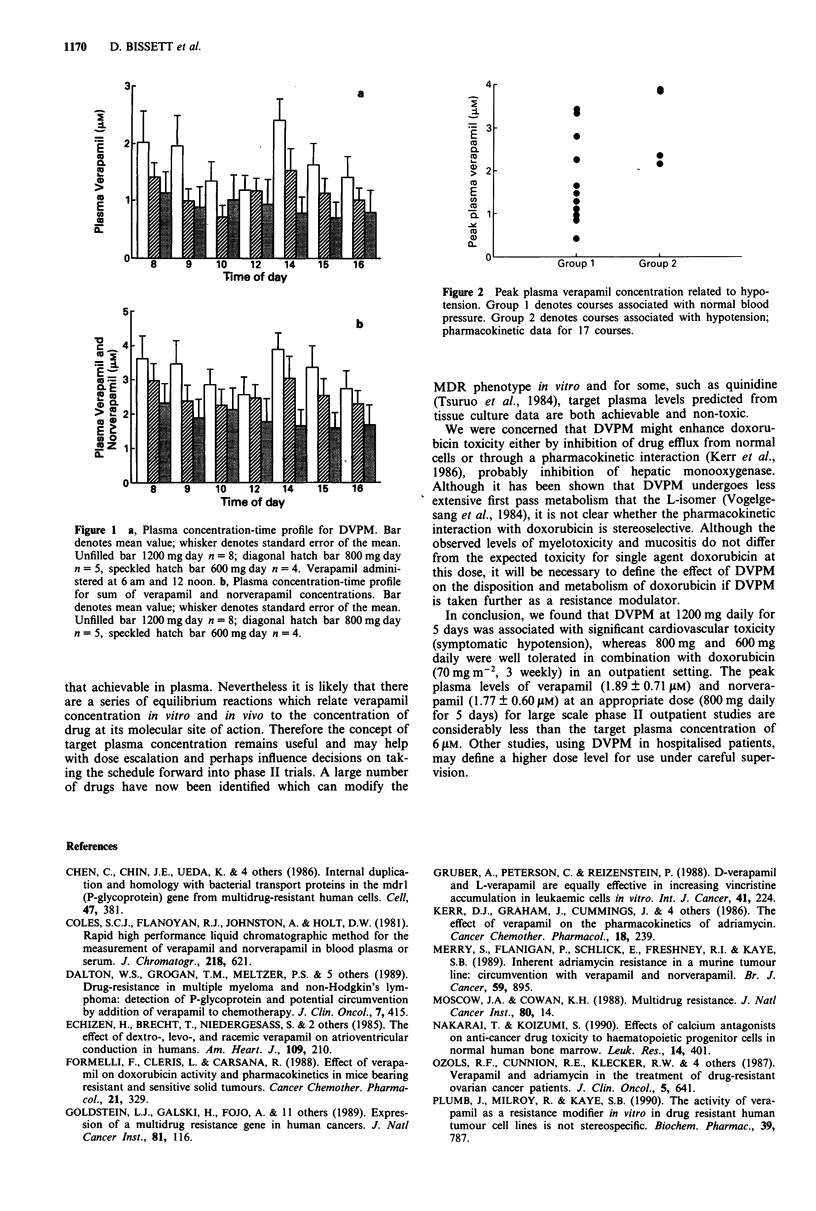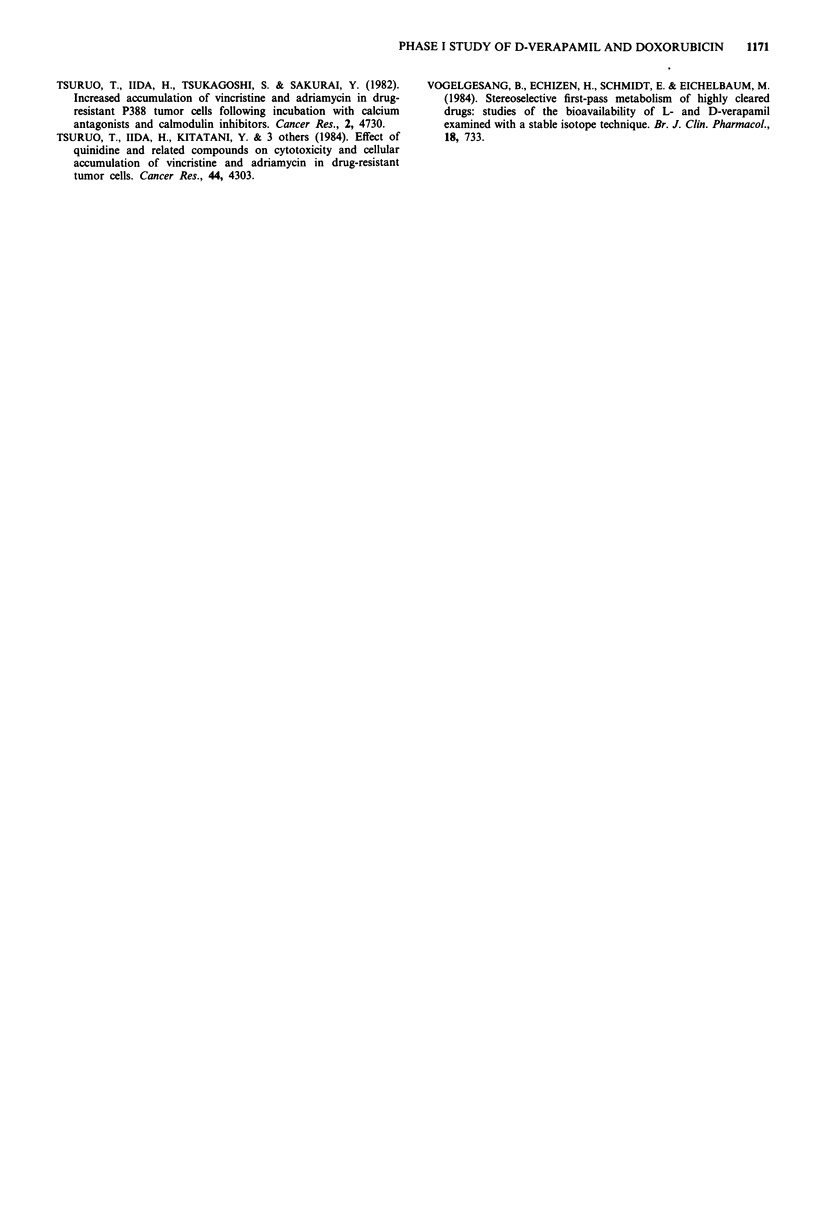# Phase I and pharmacokinetic study of D-verapamil and doxorubicin.

**DOI:** 10.1038/bjc.1991.484

**Published:** 1991-12

**Authors:** D. Bissett, D. J. Kerr, J. Cassidy, P. Meredith, U. Traugott, S. B. Kaye

**Affiliations:** CRC Department of Medical Oncology, Beatson Oncology Centre, Western Infirmary, Glasgow, UK.

## Abstract

The calcium antagonist verapamil (a mixture of D- and L-racemers) is a potent modulator of the multi-drug resistance phenotype in vitro at a concentration of 6 microM. Clinical studies have shown dose-limiting toxicity of hypotension and heart block when plasma levels approach the concentrations active in vitro. Previous data indicate that the D-isomer is less cardioactive than the L-isomer but they appear to be equipotent in reversing drug resistance in vitro. In an attempt to increase plasma verapamil concentrations, we have treated ten patients (total of 27 courses) with oral D-verapamil (DVPM), 150-300 mg 6 h, and doxorubicin i.v. 70 mg m2 q 3 weeks. Hypotension (supine systolic BP less than 100 mmHg or a fall in systolic BP of greater than 30 mmHg) occurred in 5/6 patients at 1200 mg day DVPM, in 1/5 at 800 mg day, and in 1/5 at 600 mg day. PQ prolongation (greater than 0.23 s) was demonstrated in 2/5 patients at 800 mg day DVPM. Plasma levels of DVPM and its active metabolite norverapamil were measured and, combining these, levels of 3-4 microM were achieved at 1200 mg day DVPM; however this dose is likely to lead to unacceptable toxicity in the outpatient setting. Using an oral outpatient schedule of administration, an appropriate dose of DVPM is 800 mg day. This provides a combined plasma level (for VPM and DVPM) of 2-3 microM. If DVPM is to prove useful as a resistance modulator, it may require to be administered intravenously with careful inpatient monitoring and support.


*Br. J. Cancer* (1991), **64**, 1168–1171 © Macmillan Press Ltd., 1991

### Phase I and pharmacokinetic study of D-verapamil and doxorubicin

D. Bissett[1], D.J. Kerr[1], J. Cassidy[1], P. Meredith[2], U. Traugott[3] & S.B. Kaye[1]

[1]*CRC Department of Medical Oncology, Beatson Oncology Centre, Western Infirmary and Belvidere Hospital, Glasgow;* [2]*Department of Material Medica, Stobhill Hospital, Glasgow, UK; and* [3]*Knoll AG Ludwigshafen, Germany.*

**Summary** The calcium antagonist verapamil (a mixture of D- and L-racemers) is a potent modulator of the multi-drug resistance phenotype *in vitro* at a concentration of 6 μM. Clinical studies have shown dose-limiting toxicity of hypotension and heart block when plasma levels approach the concentrations active *in vitro*. Previous data indicate that the D-isomer is less cardioactive than the L-isomer but they appear to be equipotent in reversing drug resistance *in vitro*. In an attempt to increase plasma verapamil concentrations, we have treated ten patients (total of 27 courses) with oral D-verapamil (DVPM), 150–300 mg 6 h, and doxorubicin i.v. 70 mg m2 q 3 weeks. Hypotension (supine systolic BP $<$ 100 mmHg or a fall in systolic BP of $>$ 30 mmHg) occurred in 5/6 patients at 1200 mg day DVPM, in 1/5 at 800 mg day, and in 1/5 at 600 mg day. PQ prolongation ($>$ 0.23 s) was demonstrated in 2/5 patients at 800 mg day DVPM. Plasma levels of DVPM and its active metabolite norverapamil were measured and, combining these, levels of 3–4 μM were achieved at 1200 mg day DVPM; however this dose is likely to lead to unacceptable toxicity in the outpatient setting. Using an oral outpatient schedule of administration, an appropriate dose of DVPM is 800 mg day. This provides a combined plasma level (for VPM and DVPM) of 2–3 μM. If DVPM is to prove useful as a resistance modulator, it may require to be administered intravenously with careful inpatient monitoring and support.

Resistance to cytotoxic agents is a common cause of failure of therapy in both solid tumours and haematological malignancies and there is evidence that expression of the multidrug resistance (MDR) phenotype underlies drug resistance in some tumours (Goldstein *et al.*, 1989). Although several years have elapsed since the observation that verapamil reduces resistance to vincristine and doxorubicin in certain cell lines (Tsuruo *et al.*, 1982), its role in clinical oncology is still uncertain. The modulation of drug resistance by verapamil appears independent of calcium channel blockage (Gruber *et al.*, 1988) and is at least in part due to its binding to the P170 glycoprotein with reduction of drug efflux from cells via this energy dependent pump (Chen *et al.*, 1986; Moscow *et al.*, 1988). *In vitro* studies show a dose response relationship with maximal reduction of resistance with 6 μM verapamil (Plumb *et al.*, 1990) but clinical experience of verapamil in combination with chemotherapy has shown that plasma verapamil levels of 6 μM and above are associated with hypotension and heart block (Ozols *et al.*, 1987; Dalton *et al.*, 1989) and therefore necessitate treatment in an intensive therapy unit.

P-glycoprotein is present in normal tissues and it is possible that the combination of a modulator of drug efflux and a cytotoxic agent might increase normal tissue damage. There is evidence of such enhanced damage when normal bone marrow stem cells are treated *in vitro* with verapamil and doxorubicin (Nakarai *et al.*, 1990), but preclinical *in vivo* studies have been inconclusive (Formelli *et al.*, 1988). Clearly a randomised clinical trial comparing cytotoxic treatment with and without a modulator of MDR is required to determine whether such modulation improves treatment outcome or produces more toxicity. High dose verapamil therapy with intensive support is not feasible for such a study, which should preferably comprise outpatient therapy.

Verapamil is usually prescribed as a racemic mixture but the pharmacological properties of its isomers are quite different. L-verapamil is ten times more cardioactive as measured by PR prolongation and is less protein bound to albumin (L-88%, D-93%) (Echizen *et al.*, 1985). The isomers are however equipotent as modulators of drug resistance *in vitro* (Tsuruo *et al.*, 1982; Plumb *et al.*, 1990). Predicting that the reduced cardioactivity of the d-isomer would allow safe elevation of the plasma level of verapamil towards the target 6 μM, we undertook a phase I study of DVPM in combination with doxorubicin. Our aims were to establish the maximum tolerated dose of DVPM in this combination in an outpatient setting, to define dose-limiting toxicity, and to study the pharmacokinetics of DVPM.

Correspondence: D. Bissett, University Department of Medical Oncology, Beatson Oncology Centre, Western Infirmary, Dumbarton Road, Glasgow G11 6NT, UK.
Received 22 April 1991; and in revised form 28 August 1991.

### Materials and methods

DVPM was supplied by Knoll AG (Ludwigshafen, Germany). A preliminary single dose study was undertaken in eight healthy normotensive volunteers; the first four received placebo, 240 mg racemic verapamil, and 250 mg and 500 mg DVPM, in random order at weekly intervals; the second four were treated similarly but with 500 and 1000 mg DVPM. Similar falls in blood pressure and prolongation of PQ interval were observed with 500 mg DVPM and 240 mg racemic verapamil. While 500 mg DVPM was well tolerated, 1,000 mg DVPM produced symptomatic hypotension and epigastric discomfort. Pharmacokinetic modelling using data obtained from these single dose studies suggested that oral DVPM, 300 mg given 6 h, would achieve plasma verapamil levels of about 4 μM (unpublished observations, P. Meredith).

We selected for study patients with advanced or metastatic gastric, colorectal, or renal carcinoma as these tumours can have high levels of MDR1 mRNA without prior exposure to chemotherapy (Goldstein *et al.*, 1989). Each patient had an Eastern Cooperative Oncology Group (ECOG) performance status of two or better. A minimum of 8 weeks had elapsed since prior chemotherapy and no patients had previously received anthracyclines. Other criteria for inclusion were: baseline total white cell count $> 4 \times 10^9 l^{-1}$, platelets $> 100 \times 10^9 l^{-1}$, normal serum bilirubin, and other liver function tests less than twice the upper normal limit. All patients had normal electrocardiograms with PQ intervals $<$ 0.2 s, heart rate $>$ 50 beats per min, resting systolic BP $>$ 110 mmHg, and a normal pretreatment echocardiogram; patients with a history of cardiovascular disease were excluded from the study. Written informed consent was obtained from each patient according to the dictates of the local ethical committee. Pretreatment evaluation included a history and physical examination with documentation of measurable disease when appropriate.

Oral DVPM was taken 6 h for 3 days prior to doxorubicin and continued for a further seven doses after chemotherapy. ECGs were recorded prior to and 2 h after a dose of DVPM on the 1st and 4th days of treatment and doxorubicin 70 mg $m^{-2}$ given by bolus i.v. injection on the 4th days of DVPM. Treatment was repeated every 3 weeks. Blood pressure and pulse rate were monitored in hospital on days 1 and 4 but patients remained at home for the rest of the study. Toxicity was charted using standard WHO criteria. Hypotension was defined as supine systolic BP < 100 mmHg or a fall in systolic BP of > 30 mmHg. PQ prolongation was defined as PQ interval > 0.23 s. Patients were to be withdrawn from the study if there was evidence of disease progression, grade 3 or 4 toxicity, unpredictable and life-threatening toxicity, or if the patient refused further treatment. Patients experiencing cardiovascular toxicity as defined above were subsequently treated at a reduced dose of DVPM; patients with myelosuppression of grade 3 or 4 were allowed to continue on study at the discretion of the clinician with a reduction in the dose of doxorubicin to 50 mg $m^{-2}$. Blood samples for DVPM pharmacokinetics were taken at 2, 3, 4, 6, 8, 9 and 10 h after the first dose of DVPM on the 4th day. Verapamil and norverapamil levels were measured by a sensitive and specific HPLC method with fluorescent detection (Cole *et al.*, 1981); the assay does not differentiate between the D- and L- isomers. The intra-assay and the inter-assay coefficients of variation were less than 5% for both verapamil and norverapamil. The limit of detection of the assay was 2 ng $ml^{-1}$ using a 100 µl plasma sample.

### Results

Ten patients were entered into the study and received a total fo 27 courses of treatment. Their details are summarised in Table I and the pattern of toxicity is shown in Table II. The starting dose of DVPM was chosen on the basis of the normal volunteer study. The first six patients entered the study at a daily dose of 1200 mg DVPM but 5/6 developed hypotension (supine systolic BP < 100 mmHg and/or a drop in systolic BP > 30 mmHg) during the first or second course of DVPM and three continued on study at 800 mg day DVPM. Two additional patients were entered at 800 mg day. PQ prolongation (> 0.23 s) was not seen at 1200 mg day DVPM but did occur in 2/5 patients receiving 800 mg day. Hypotension was observed in 1/5 at 800 mg day and the three patients with toxicity at this dose received further DVPM at 600 mg day. Two additional patients were entered at this dose but one became hypotensive during the first course. Three patients at 1200 mg day with hypotension were symptomatic, but none at 800 mg and 600 mg. All cardiovascular toxicity reversed spontaneously on cessation of DVPM and no specific pressor therapy was required to raise blood pressure.

Gastro-intestinal toxicity was frequent but mild: nausea and vomiting grade 0 in 2/10, grade 1 in 4/10 and grade 2 in 4/10 patients; and oral mucositis grade 0 in 4/10, grade 1 in 2/10, grade 2 in 3/10, and grade 3 in 1/10 patients. Myelotoxicity of grade 3 or 4 occurred in 4/10 patients, leading to subsequent reduction of the doxorubicin dose to 50 mg $m^{-2}$ in two patients.

One patient with gastric cancer and a large paraaortic node mass had a partial response, documented on CT scan, of 8 months duration. No other evidence of response was documented.

**Table I** Summary of patient details

| | | |
|---|---|---|
| Number of patients | | 10 |
| Age (years) | Median | 49 |
| | Range | 44–62 |
| Sex | Male | 3 |
| | Female | 7 |
| Performance status (ECOG) | | 0–4 patients<br>1–6 patients |
| Primary tumour | | 3 stomach<br>4 colon or rectum<br>2 adenocarcinoma of unknown primary<br>1 kidney |
| Extent of disease | | 6 hepatic metastases<br>1 inoperable primary<br>1 pulmonary and paraaortic lymphadenopathy<br>1 peritoneal metastases<br>1 post-gastrectomy node positive |
| Number of courses | Median | 3 |
| | Range | 1–5 |

Plasma levels of both verapamil and norverapamil showed considerable inter-patient variation (Figure 1a and b) and patients who developed hypotension tended to have higher peak plasma DVPM levels than patients who remained normotensive ($P = 0.04$, Mann Whitney U-test) (Figure 2). When the parent compound and active metabolite (Merry *et al.*, 1989) levels are combined, levels of 3–4 µM are achieved at a daily dose of 1200 mg DVPM.

### Discussion

The target plasma level of verapamil predicted by *in vitro* studies of resistance modulation is 6 µM and we have observed cardiovascular toxicity at half this level with DVPM. Although the volunteer study showed equivalent cardiovascular activity with 500 mg DVPM and 240 mg racemic verapamil, DVPM drug has shown more cardiovascular toxicity than predicted (Echizen *et al.*, 1985) and we cannot adequately explain this. It is known that verapamil can inhibit its own hepatic clearance but the plasma levels measured in patients at 1200 mg day were actually lower than predicted from the volunteer study. A possible explanation would be *in vivo* alteration of chirality but there is no evidence of this in single dose studies of DVPM (Vogelgesang *et al.*, 1984); a chiral assay for verapamil has not yet been developed. It appears unlikely that levels of 6 µM will be achieved safely in outpatient treatment regimens including DVPM as a resistance modulator although this may not be the case if DVPM is used on an inpatient basis with careful supervision and perhaps pressor support. Certainly DVPM is less cardioactive than racemic VPM and should be the preferred agent if high plasma concentrations of verapamil are sought.

The relevance of 6 µM plasma levels to modulation of drug resistance in clinical practice remains speculative. As yet we do not know how levels of verapamil achieved in tumour *in vivo* relate to plasma concentrations; nor do we know what VPM concentration in tumour is necessary to modulate clinical multi-drug resistance. Given the extent of protein binding of verapamil it is difficult to know what relationship exists between the concentration of verapamil active *in vitro* and

**Table II** Pattern of toxicity observed at each dose level of DVPM

| *Number of patients* | *Dose DVPM (mg day)* | *Number of courses* | *PQ prolongation (> 0.23 s)* | *Hypotension (systolic BP < 100 mmHg or fall of > 30 mmHg* | *Myelotoxicity WHO Grade* | | | | | *Nausea and vomiting WHO Grade* | | | | *Oral mucositis WHO Grade* | | | |
|---|---|---|---|---|---|---|---|---|---|---|---|---|---|---|---|---|---|
| | | | | | *0* | *1* | *2* | *3* | *4* | *0* | *1* | *2* | *3* | *0* | *1* | *2* | *3* |
| 6 | 1200 | 9 | 0 | 5 | 1 | 1 | 4 | 0 | 0 | 3 | 1 | 2 | 0 | 5 | 0 | 1 | 0 |
| 5 | 800 | 8 | 2 | 1 | 0 | 1 | 2 | 1 | 1 | 0 | 3 | 2 | 0 | 2 | 2 | 1 | 0 |
| 5 | 600 | 10 | 0 | 1 | 0 | 1 | 0 | 1 | 3 | 2 | 3 | 0 | 0 | 1 | 1 | 2 | 1 |

**Figure 1** **a**, Plasma concentration-time profile for DVPM. Bar denotes mean value; whisker denotes standard error of the mean. Unfilled bar 1200 mg day $n = 8$; diagonal hatch bar 800 mg day $n = 5$, speckled hatch bar 600 mg day $n = 4$. Verapamil administered at 6 am and 12 noon. **b**, Plasma concentration-time profile for sum of verapamil and norverapamil concentrations. Bar denotes mean value; whisker denotes standard error of the mean. Unfilled bar 1200 mg day $n = 8$; diagonal hatch bar 800 mg day $n = 5$, speckled hatch bar 600 mg day $n = 4$.

**Figure 2** Peak plasma verapamil concentration related to hypotension. Group 1 denotes courses associated with normal blood pressure. Group 2 denotes courses associated with hypotension; pharmacokinetic data for 17 courses.

that achievable in plasma. Nevertheless it is likely that there are a series of equilibrium reactions which relate verapamil concentration *in vitro* and *in vivo* to the concentration of drug at its molecular site of action. Therefore the concept of target plasma concentration remains useful and may help with dose escalation and perhaps influence decisions on taking the schedule forward into phase II trials. A large number of drugs have now been identified which can modify the MDR phenotype *in vitro* and for some, such as quinidine (Tsuruo *et al.*, 1984), target plasma levels predicted from tissue culture data are both achievable and non-toxic.

We were concerned that DVPM might enhance doxorubicin toxicity either by inhibition of drug efflux from normal cells or through a pharmacokinetic interaction (Kerr *et al.*, 1986), probably inhibition of hepatic monooxygenase. Although it has been shown that DVPM undergoes less extensive first pass metabolism that the L-isomer (Vogelgesang *et al.*, 1984), it is not clear whether the pharmacokinetic interaction with doxorubicin is stereoselective. Although the observed levels of myelotoxicity and mucositis do not differ from the expected toxicity for single agent doxorubicin at this dose, it will be necessary to define the effect of DVPM on the disposition and metabolism of doxorubicin if DVPM is taken further as a resistance modulator.

In conclusion, we found that DVPM at 1200 mg daily for 5 days was associated with significant cardiovascular toxicity (symptomatic hypotension), whereas 800 mg and 600 mg daily were well tolerated in combination with doxorubicin ($70 \text{ mg m}^{-2}$, 3 weekly) in an outpatient setting. The peak plasma levels of verapamil ($1.89 \pm 0.71$ μM) and norverapamil ($1.77 \pm 0.60$ μM) at an appropriate dose (800 mg daily for 5 days) for large scale phase II outpatient studies are considerably less than the target plasma concentration of 6 μM. Other studies, using DVPM in hospitalised patients, may define a higher dose level for use under careful supervision.

### References

CHEN, C., CHIN, J.E., UEDA, K. & 4 others (1986). Internal duplication and homology with bacterial transport proteins in the mdr1 (P-glycoprotein) gene from multidrug-resistant human cells. *Cell*, **47,** 381.

COLES, S.C.J., FLANOYAN, R.J., JOHNSTON, A. & HOLT, D.W. (1981). Rapid high performance liquid chromatographic method for the measurement of verapamil and norverapamil in blood plasma or serum. *J. Chromatogr.*, **218,** 621.

DALTON, W.S., GROGAN, T.M., MELTZER, P.S. & 5 others (1989). Drug-resistance in multiple myeloma and non-Hodgkin's lymphoma: detection of P-glycoprotein and potential circumvention by addition of verapamil to chemotherapy. *J. Clin. Oncol.*, **7,** 415.

ECHIZEN, H., BRECHT, T., NIEDERGESASS, S. & 2 others (1985). The effect of dextro-, levo-, and racemic verapamil on atrioventricular conduction in humans. *Am. Heart. J.*, **109,** 210.

FORMELLI, F., CLERIS, L. & CARSANA, R. (1988). Effect of verapamil on doxorubicin activity and pharmacokinetics in mice bearing resistant and sensitive solid tumours. *Cancer Chemother. Pharmacol.*, **21,** 329.

GOLDSTEIN, L.J., GALSKI, H., FOJO, A. & 11 others (1989). Expression of a multidrug resistance gene in human cancers. *J. Natl Cancer Inst.*, **81,** 116.

GRUBER, A., PETERSON, C. & REIZENSTEIN, P. (1988). D-verapamil and L-verapamil are equally effective in increasing vincristine accumulation in leukaemic cells *in vitro*. *Int. J. Cancer*, **41,** 224.

KERR, D.J., GRAHAM, J., CUMMINGS, J. & 4 others (1986). The effect of verapamil on the pharmacokinetics of adriamycin. *Cancer Chemother. Pharmacol.*, **18,** 239.

MERRY, S., FLANIGAN, P., SCHLICK, E., FRESHNEY, R.I. & KAYE, S.B. (1989). Inherent adriamycin resistance in a murine tumour line: circumvention with verapamil and norverapamil. *Br. J. Cancer*, **59,** 895.

MOSCOW, J.A. & COWAN, K.H. (1988). Multidrug resistance. *J. Natl Cancer Inst.*, **80,** 14.

NAKARAI, T. & KOIZUMI, S. (1990). Effects of calcium antagonists on anti-cancer drug toxicity to haematopoietic progenitor cells in normal human bone marrow. *Leuk. Res.*, **14,** 401.

OZOLS, R.F., CUNNION, R.E., KLECKER, R.W. & 4 others (1987). Verapamil and adriamycin in the treatment of drug-resistant ovarian cancer patients. *J. Clin. Oncol.*, **5,** 641.

PLUMB, J., MILROY, R. & KAYE, S.B. (1990). The activity of verapamil as a resistance modifier *in vitro* in drug resistant human tumour cell lines is not stereospecific. *Biochem. Pharmac.*, **39,** 787.

TSURUO, T., IIDA, H., TSUKAGOSHI, S. & SAKURAI, Y. (1982). Increased accumulation of vincristine and adriamycin in drug-resistant P388 tumor cells following incubation with calcium antagonists and calmodulin inhibitors. *Cancer Res.*, **2**, 4730.

TSURUO, T., IIDA, H., KITATANI, Y. & 3 others (1984). Effect of quinidine and related compounds on cytotoxicity and cellular accumulation of vincristine and adriamycin in drug-resistant tumor cells. *Cancer Res.*, **44**, 4303.

VOGELGESANG, B., ECHIZEN, H., SCHMIDT, E. & EICHELBAUM, M. (1984). Stereoselective first-pass metabolism of highly cleared drugs: studies of the bioavailability of L- and D-verapamil examined with a stable isotope technique. *Br. J. Clin. Pharmacol.*, **18**, 733.